# Oligodendrocyte precursor cell-derived exosomes combined with cell therapy promote clinical recovery by immunomodulation and gliosis attenuation

**DOI:** 10.3389/fncel.2024.1413843

**Published:** 2024-07-23

**Authors:** Sarah Ingrid Pinto Santos, Santiago José Ortiz-Peñuela, Alessandro de Paula Filho, Ana Laura Midori Rossi Tomiyama, Lilian de Oliveira Coser, Juliano Coelho da Silveira, Daniele dos Santos Martins, Adriano Polican Ciena, Alexandre Leite Rodrigues de Oliveira, Carlos Eduardo Ambrósio

**Affiliations:** ^1^Faculty of Animal Science and Food Engineering, University of São Paulo (FZEA/USP), São Paulo, Brazil; ^2^Institute of Biology, University of Campinas (IB/UNICAMP), Campinas, Brazil; ^3^Institute of Biosciences, São Paulo State University, Rio Claro, Brazil

**Keywords:** extracellular vesicles, multiple sclerosis, neural stem cell, neurodegeneration, immunomodulation, oligodendrocyte precursor cell

## Abstract

Multiple sclerosis is a chronic inflammatory disease of the central nervous system characterized by autoimmune destruction of the myelin sheath, leading to irreversible and progressive functional deficits in patients. Pre-clinical studies involving the use of neural stem cells (NSCs) have already demonstrated their potential in neuronal regeneration and remyelination. However, the exclusive application of cell therapy has not proved sufficient to achieve satisfactory therapeutic levels. Recognizing these limitations, there is a need to combine cell therapy with other adjuvant protocols. In this context, extracellular vesicles (EVs) can contribute to intercellular communication, stimulating the production of proteins and lipids associated with remyelination and providing trophic support to axons. This study aimed to evaluate the therapeutic efficacy of the combination of NSCs and EVs derived from oligodendrocyte precursor cells (OPCs) in an animal model of multiple sclerosis. OPCs were differentiated from NSCs and had their identity confirmed by gene expression analysis and immunocytochemistry. Exosomes were isolated by differential ultracentrifugation and characterized by Western, transmission electron microscopy and nanoparticle tracking analysis. Experimental therapy of C57BL/6 mice induced with experimental autoimmune encephalomyelitis (EAE) were grouped in control, treated with NSCs, treated with OPC-derived EVs and treated with a combination of both. The treatments were evaluated clinically using scores and body weight, microscopically using immunohistochemistry and immunological profile by flow cytometry. The animals showed significant clinical improvement and weight gain with the treatments. However, only the treatments involving EVs led to immune modulation, changing the profile from Th1 to Th2 lymphocytes. Fifteen days after treatment revealed a reduction in reactive microgliosis and astrogliosis in the groups treated with EVs. However, there was no reduction in demyelination. The results indicate the potential therapeutic use of OPC-derived EVs to attenuate inflammation and promote recovery in EAE, especially when combined with cell therapy.

## 1 Introduction

Multiple sclerosis (MS) is an inflammatory disease of the central nervous system (CNS) characterized by autoimmune destruction of the myelin sheath and neuroinflammation. The demyelination promoted by MS exposes the axons and makes them prone to injury and degenerative processes. These disorders trigger severe neurological symptoms related to sensory, motor and cognitive dysfunctions that incapacitate the patient (Lucchinetti et al., 2000).

Experimental autoimmune encephalomyelitis (EAE) is the most widely used animal model of MS due to its similarity to the pathophysiology of the disease. The mechanisms that hinder the resolution of the inflammatory process and the success of remyelination in multiple sclerosis are also mimicked in the EAE model, which allows experimental therapies to be better explored. Mice are induced to develop EAE through immunizations that sensitize them to myelin antigens. This sensitization occurs through adjuvants containing bacterial components with a high potential for activating the innate immune system (Constantinescu et al., 2011).

Under physiological conditions, oligodendrocyte precursor cells (OPC) and neural stem cells (NSC) act in the central nervous system by differentiating into mature oligodendrocytes that remyelinate demyelinated axons. However, within the pathophysiological mechanism of MS and EAE, the regenerative capacity of these cells is quite limited and inefficient, leading to progressive and permanent neurodegeneration (Gruchot et al., 2019). Following this principle, stem cell-based therapies have been evaluated as a possible therapeutic approach to modulate the inefficient immune system, regenerate injured nerve tissue and stimulate remyelination (Scolding et al., 2017).

Neural stem cells have the ability to differentiate into neuronal and glial cells, as well as release neurotrophic factors that promote neurogenesis in the central nervous system (Alessandrini et al., 2019). In a phase I clinical study carried out by Harris et al. (2018), MS patients were treated with mesenchymal stem cell-derived NSCs and there was an improvement in neurological disability in 70% of patients, especially in terms of motor function. However, despite the positive results observed, therapy with stem cells alone has not yet been able to reach satisfactory therapeutic levels.

At this point, extracellular vesicles (EVs) emerge as important therapeutic mediators that act in intercellular communication through the transport of genetic material and proteins (Osorio-Querejeta et al., 2018). Among the types of EVs, exosomes are the most explored due to their intrinsic characteristics, such as stability, biocompatibility and ability to cross the blood–brain barrier. Some important components secreted by stem cells and which have therapeutic properties for neurodegenerative diseases have been discovered to be some of the elements carried by their exosomes (Lai et al., 2010). Studies then began to evaluate the therapeutic potential of exosomes isolated from these cells and observed that the therapeutic use of stem cell-derived exosomes in various diseases was able to attenuate inflammation, promote neurogenesis, angiogenesis and tissue regeneration, consequently resulting in significant clinical improvement (Teng et al., 2015; Huang et al., 2017; Lee et al., 2023).

Extracellular vesicles derived from mature oligodendrocytes (EV-OL), however, were able to modulate the immune system in order to stimulate immune tolerance in different models of EAE (induced with the antigens MOG, MBP and PLP). In these three models, EVs were able to induce the activity of immunosuppressive monocytes and the apoptosis of self-reactive CD4+ lymphocytes. EV-OPC and EV-OL protein content is quite different, with the absence of myelin proteins in EV-OPC (Casella et al., 2020). This is the only recent data in the literature on extracellular vesicles derived from oligodendrocyte in EAE and oligodendrocyte precursor cells content. Knowledge about EVs derived from these precursor cells could elucidate the mechanisms of intercellular communication during the myelination/remyelination process, as well as acting as a new tool within therapeutic protocols of MS and other demyelinating diseases. The ability to transport bioactive proteins and genetic material makes the exosomes of oligodendrocyte precursor cells a tool with high potential for increasing the efficiency of cell therapy in EAE and, therefore, should be better understood.

## 2 Materials and methods

### 2.1 Isolation and culture of neural stem cells

Neural stem cells (NSC) were isolated from the subventricular zone of neonatal C57BL/6 mice, following a procedure described by Guo et al. (2012). Single-cells isolated were cultured in a NSC growth medium consisting of DMEM/F12 supplemented with 2% B-27 minus vitamin A (Gibco), 1% L-glutamine (Gibco), 1% penicillin/streptomycin (Gibco), 20 ng/mL EGF (Peprotech), and 20 ng/mL bFGF (Peprotech). Over a 7-day culture period, single cells formed neurospheres, which were then re-plated onto an adherent culture system in flasks treated with 20 μg/mL Poly-L-Ornithine (PLO) (Sigma).

### 2.2 Differentiation to oligodendrocyte precursor cells

The differentiation followed the protocol outlined by Li et al. (2019). NSC were cultured at a concentration of 4 × 10^4^ cells/cm^2^ for 24 h in culture flasks pre-treated with PLO for 24 h to allow cell adherence. Subsequently, the NSC growth medium was replaced with a differentiation medium for oligodendrocyte precursor cells (OPCs). This medium consisted of DMEM/F12 supplemented with 1% B-27 (Gibco), 1% N-2 (Gibco), 1% penicillin/streptomycin (Gibco), 20 ng/mL bFGF (PeproTech), 20 ng/mL PDGF-AA (PeproTech), and 0.4 μM SAG (Sigma-Aldrich). The medium change was performed every 48 h.

### 2.3 Immunocytochemistry

NSC and OPC were fixed with 4% paraformaldehyde (PFA) for 30 min at room temperature and washed three times with PBS for 5 min each wash. Following fixation, the cells were permeabilized in 0.2% Triton X-100 (Invitrogen) for 15 min at room temperature and then washed three times with PBS for 5 min each. Blocking was performed with 1% bovine serum albumin (BSA) for 60 min, after which the cells were incubated with the primary antibodies overnight at 4°C (Table 1). The primary antibodies used to characterize NSC were anti-Nestin (Merck Millipore cat# MAB353, conc. 1:200), anti-SOX2 (Sigma-Aldrich cat# S9072, conc. 1:200), anti-β-Tubulin III (Merck Millipore cat# MAB1637, conc. 1:500) and anti-GFAP (Merck Millipore cat# AB5804, conc. 1:1000) and to characterize OPC were anti-NG2 (Invitrogen cat# MA5-24247, conc. 1:300), anti-PDGFRA (Merck millipore cat# PA5-16571, conc. 1:500) and anti-MBP (Abcam cat# ab7349, conc. 1:200). Cells were washed with PBS and then incubated with fluorescent secondary antibodies Alexa 488 (Invitrogen cat# A-21042, conc. 1:800) and Alexa 568 (Invitrogen cat# A-11011, conc. 1:800) for 1 h at room temperature. Nuclei were stained with 0.1 μg/mL DAPI (Sigma) for 5 min. Immunolabeled cells were evaluated using the EVOS M5000 Imaging System fluorescence microscope (Life Technologies). Five random fields from NG2 and PDGFRA immunolabeled OPCs were used to quantify the number of positive cells in relation to the DAPI-labeled nucleus to validate the homogeneity of the cell population.

**Table 1:** Antibodies used for immunocytochemistry analysis of NSC and OPC.

**Antibodies**	**Host**	**Source (RRID)**	**Dilution**
NESTIN	Mouse	Merck Millipore (AB_94911)	1:200
SOX-2	Rabbit	Merck Millipore (AB_1841175)	1:200
GFAP	Rabbit	Merck Millipore (AB_2109645)	1:1000
TUJ1	Mouse	Merck Millipore (AB_2210524)	1:500
PDGFRa	Rabbit	Merck Millipore (AB_10981626)	1:500
NG2	Mouse	Invitrogen (AB_2606388)	1:300
MBP	Rat	Abcam (AB_305869)	1:200
Alexa488	Mouse	Invitrogen (AB_2535711)	1:800
Alexa568	Rabbit	Invitrogen (AB_143157)	1:800

### 2.4 NSC and OPC gene expression analysis

Gene expression analysis was performed by RT-qPCR. Total RNA was extracted from 1 × 10^6^ NSC and differentiated OPC to assess the expression levels of specific genes associated with neural stem cells, neural precursor cells and oligodendrocyte precursor cells using the TRIzol method (Thermo Fisher) following the manufacturer’s instructions. The isolated RNA was purified with the RNeasy Plus Universal kit (QIAGEN) until obtaining a 30 μL sample. The success of the extraction was verified by Nanodrop 1,000 spectrophotometry (Thermo Scientific), assessing the concentration and quality of the RNA sample. For conversion, 1,000 ng/μL of total RNA and the High-Capacity cDNA Reverse Transcription kit (Applied Biosystems) were used according to the manufacturer’s instructions. The Veriti thermocycler (Applied Biosystems) was set to the following conditions for conversion: 25°C for 10 min, 37°C for 120 min, 85°C for 5 min, and 4°C until the sample removal and storage.

For qPCR, were used primers described by Otsu et al. (2021), that can be viewed in Table 2, for genes *NESTIN, HES1, ASCL1* (neural stem cells/neural progenitors), *GFAP, TUJ1, OLIG1* (neural precursor cells), *PDGFRA, PLP1, NKX2.2, SOX-10* (oligodendrocyte lineage) *and GAPDH* (endogenous control). Reactions were performed using the SYBR Green Master Mix (Applied Biosystems), specific primers for each studied gene and 20 ng/μL of the sample. The StepOne thermocycler (Applied Biosystems) was set to the following conditions: 94°C for 10 min before starting 40 cycles of 94°C for 30 s and 60°C for 1 min, followed by a melting curve under the same parameters. The results were analyzed in technical duplicate and biological triplicate using the 2^-ΔCt^ method normalized to the expression of the endogenous control gene GAPDH.

**Table 2:** Sequence of qPCR primers specific to mice.

**Target gene** **(Access number - Genbank)**	**Primer sequence (5′ -- 3′)**	**Product size (bp)**
Neural stem cell markers		
NESTIN (NM_016701.3)	F: AGTGCCCAGTTCTACTGGTGTCC R: CCTCTAAAATAGAGTGGTGAGGGTTGA	124
HES1 (NM_008235.2)	F: ACGGCCAATTTGCCTTTCT R: GGAAGGTGACACTGCGTTAGG	115
ASCL1 (NM_008553.5)	F: TCCTGTCGCCCACCATCT R: TGGGCTAAGAGGGTCGTAGGAT	109
Neural precursor cell markers		
TUJ1 (NM_023279.3)	F: CTTTATCTTCGGTCAGAGTGGTGC R: TTCTTTCCGCACGACATCTAGG	101
*GFAP* (NM_010277.3)	F: CGTTAAGCTAGCCCTGGACATC R: GGATCTGGAGGTTGGAGAAAGTC	106
*OLIG1* (NM_016968.4)	F: AGGCAGCCACCTATCTCCTCA R: AGCGGAGCTTCGGCCTT	91
Oligodendrocyte lineage markers		
NKX2.2 (NM_010919.2)	F: TCAGTCAAGGACATCTTGGACCT R: TTCGCTCTCCTCCTCTGGC	80
PDGFRα (NM_011058.3)	F: CCATCGAGACAGGTTCCAGTAGT R: GGTCCGAGGAATCTATACCAATGT	101
SOX-10 (NM_011437.1)	F: TCACGACCCCAGTTTGACTATTC R: CCCCATGTAAGAAAAGGCTGAA	101
PLP1 (NM_011123.4)	F: TGGCACTGTTCTGTGGATGTG R: GTCCTGGTAGTTTTTGGAGAAATAGGT	86
Endogenous marker		
*GAPDH* (NM_008084.3)	F: CTGCACCACCAACTGCTTAGC R: CAGTCTTCTGGGTGGCAGTGA	109

F, Forward; R, Reverse. Adapted from Otsu et al. (2021).

### 2.5 Isolation of OPC-derived exosomes

Exosomes were isolated by differential ultracentrifugation. The conditioned medium from differentiated OPC was collected and submitted to serial centrifugations for clarification, aiming to purify the content of extracellular vesicles (EVs) and eliminate debris. The first centrifugation was performed for 10 min at 300 × *g* to remove floating cells, the second for 10 min at 2,000 × *g* to eliminate cellular debris and the third for 30 min at 16,500 × *g* to remove intracellular organelles. Subsequently, the clarified conditioned medium was filtered through a 0.2 μm syringe filter to retain larger EVs and small extracellular vesicles were isolated by ultracentrifugation using the Optima XE-90 Ultracentrifuge (Beckman Coulter). The ultracentrifugation process occurred in two steps. The first step involved centrifugation at 119,700 × *g* for 70 min to remove the culture medium and replace it with PBS for sample washing. The second step involved further centrifugation for 70 min at 119,700 × *g* to discard PBS and obtain the EV pellet. All centrifugation and ultracentrifugation steps were conducted at 4°C. The isolated exosomes for characterization were immediately used while the exosomes intended for treatment were stored in a liquid nitrogen tank.

### 2.6 Nanoparticle tracking analysis

The size and concentration of exosomes derived from OPC (EV-OPC) were determined using nanoparticle tracking analysis (NTA) with the Nanosight NS300 equipment (Malvern Panalytical) and NanoSight NTA v3.1 software. EVs isolated were resuspended in 40 μL of PBS and then diluted to a concentration of 1:100 in PBS. Next, 500 μL of the diluted sample was injected into the Nanosight equipment for analysis. NTA was performed with a 405 nm laser, camera level of ±13, a detection limit of 5 and a temperature of 37.5°C. The NanoSight NTA v.3.1 software (RRID: SCR_014239) was configured to capture 5 videos, each with a duration of 30 s per sample. This analysis allowed the assessment of the homogeneity of samples isolated by differential ultracentrifugation.

### 2.7 Transmission electron microscopy

After ultracentrifugation, EVs were resuspended in fixation solution (2% glutaraldehyde, 2% paraformaldehyde, 0.1 M sodium cacodylate at pH 7.4) and kept at room temperature for 2 h. After that EVs were centrifuged at 119,700 × g for 70 min to remove the fixation solution. The formed pellet was then resuspended the buffer solution (0.1 M sodium cacodylate at pH 7.4).

Then the sample was placed on a copper grid coated with Pioloform and allowed to air-dry (approximately 30 min). Subsequently, 2% uranyl acetate was added to the grid for 3 min for staining. Excess solution was removed with a filter paper and the sample was analyzed by the JEM 100CXII transmission electron microscope (JEOL) at 80 kV.

### 2.8 Western blotting

EV-OPC proteins were extracted using RIPA 5x and OPC protein using pure RIPA. The extracted proteins were quantified using the Pierce™ BCA Protein Assay kit, following the manufacturer’s instructions. Proteins were treated with β-mercaptoethanol (Gibco) and Laemmli 4x buffer (Bio-Rad) at 95°C for 5 min. 10 μg of proteins from each sample were loaded per well onto a 4–15% Mini-PROTEAN® TGX™ Precast Protein Gel (Bio-Rad). Protein separation occurred by electrophoresis at 100 V and 20 mA for approximately 90 min. After electrophoresis, the gel was transferred using the “sandwich” system to a polyvinylidene fluoride (PVDF) membrane Trans-Blot Turbo Mini 0.2 μm (Bio-Rad) previously treated with the kit buffer for 3 min at 24 V. The membrane was blocked for 1 h in 5% non-fat milk diluted in Tris-buffered saline with Tween 20 (TBST, pH 7.4). Then, the membrane was incubated overnight with primary antibodies anti-Alix (Sigma-Aldrich cat# SAB4200476, conc. 1:1000, RRID: AB_3096066) and anti-GRP78 (Santa cruz biotechnology cat# sc-376768, conc. 1:1000, RRID: AB_2819145) at 4°C with gentle agitation. Subsequently, the membranes were washed three times with TBST for 5 min each and then incubated with the horseradish peroxidase-conjugated secondary antibody for 1 h. Proteins were detected using the Clarity™ Western ECL Substrate kit (Bio-Rad) and imaged with the ChemiDoc MP Basic Imaging System (Bio-Rad).

### 2.9 EAE induction, clinical assessment and treatment

The use of animals in this experiment was approved by the Ethics Committee on Animal Use (CEUA) of the Faculty of Animal Science and Food Engineering at the University of São Paulo under protocol number 2453090621 and by the Ethics Committee on Animal Use of the Institute of Biology at the State University of Campinas under protocol number 61831/2023. Twenty-four 10-week-old female C57BL/6 mice weighing 18-20 g were housed in groups of three per cage, temperature-controlled under a 12-h light/dark cycle, receiving a standard diet consisting of pelletized feed and mineral water *ad libitum*. For EAE induction, the animals were anesthetized by inhalation with isoflurane to receive 100 μL of the emulsion subcutaneously in each flank. The emulsion was composed of 1 mg/mL of MOG35-55 antigen and 5 mg/mL of inactivated *Mycobacterium tuberculosis* in Freund’s adjuvant. After the emulsion has been applied, the first injection of 50 ng/μL of pertussis toxin (PTX) is made immediately via the intraperitoneal route and after 48 h the second dose of PTX via the same route. The mice were randomly distributed into 4 experimental groups. Treatment began 15 days after the induction of EAE, at the onset of the disease. Then animals were treated intravenously (lateral tail vein) and monitored for a 15 more days. The control group received the vehicle (PBS); NSC + EV group received 5 × 10^5^ cells and 2.5 × 10^9^ particles; EV group received 2.5 × 10^9^ particles only and; NSC group received 5 × 10^5^ cells only. The clinical assessment of the animals included evaluating their body weight and their clinical score daily for 30 days from EAE induction. The clinical score was determined using a scale of 0 to 5 that can be viewed in Table 3.

**Table 3:** Clinical scores for assessment of EAE-induced animals.

**Clinical Score**	**Clinical description**
0	No clinical signs
0,5	Tail tone reduction distally
1	Complete flaccid tail
1,5	Tail paralysis and walking imbalance
2	Tail paralysis and hind limb frailty (unilateral)
2,5	Tail paralysis and hind limb frailty (bilateral)
3	Hind limb paralysis (bilateral)
3,5	Hind limb paralysis (bilateral) of and fore limbs frailty
4	Paralysis of hind and fore limbs (bilateral)
5	Death

Marín‐Prida et al. (2022).

### 2.10 Flow cytometry of immune cells from spinal cord

Flow cytometry assessment of the spinal cord was performed according to Coser et al. (2024). To assess the CD4+ T lymphocyte profile in the spinal cord, three animals of each group were euthanized 72 h after the experimental treatments. The spinal cord was dissected and mechanically dissociated. Dissociated tissue was filtered twice through 140 μm filter, once through 70 μm filter and centrifuged at 400 × *g* for 10 min at 4°C. The supernatant was discarded and the pellet resuspended in 1 mL of 70% Percoll. Next, 1 mL of 37% Percoll and 1 mL of 10% Percoll were added to the tube. The Percoll samples were centrifuged at 400 × *g* for 30 min at 4°C with acceleration level 8 and deceleration level 0. After centrifugation, the myelin accumulated on the surface was discarded and the fractions carefully pipetted and separated into cytometry tubes. The fractions were centrifuged again at 400 × g for 10 min at 4°C to form the cell pellet. The pellet from each fraction was resuspended in 1 mL of DMEM culture medium supplemented with 10% SFB and 1% penicillin/streptomycin. The cells were plated in a 24-well culture plate and incubated in 250 ng/mL iomycin, 50 ng/mL PMA and 1 μg/mL and brefeldin A for 3 h in a culture incubator at 37.5°C and 5% CO2. After this stimulation stage, the cells were centrifuged and the pellet resuspended in PBS. Each fraction was placed in a 48-well microtiter plate. The cells were incubated with the conjugated antibodies in two stages (Table 4). First, they were incubated in the anti-CD4 (BioLegend cat#100423, conc. 1:50) and anti-CD3 (BioLegend cat# 100222, conc. 1:50) surface markers for 30 min at 4°C. Then, the cells were incubated overnight at 4°C with the intracellular markers anti-IFNγ (BioLegend cat#505826, conc. 1:50) and anti-IL-4 (BioLegend cat#504106, conc. 1:50). Following, cells were permeabilized and fixed in 2% PFA. The plate was read on the NovoCyte flow cytometer (Agilent).

### 2.11 Immunohistochemistry

**Figure 1:**
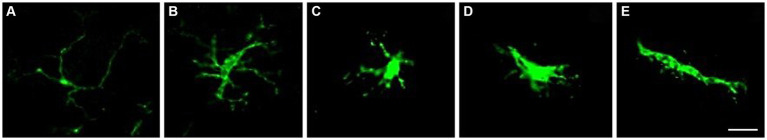
Microglia classification based on their morphology. Homeostatic microglia have long branches and a small soma **(A)**. It is important to note that hyper-ramified microglia are still considered non-reactive. Hyper-ramified microglia are at an intermediate stage between homeostatic and reactive microglia, and have long, wider branches and a larger soma **(B)**. Reactive microglia have wide, retracted branches and a larger soma **(C)**. Reactive microglia can also adopt an ameboid morphology, resembling macrophages, with minimal processes **(D)**. Additionally, some reactive microglia can take on a rod shape, with elongated soma and more extended, polarized branches **(E)**. Scale bar: 20 μm.

After 30 days, three mice of each group were euthanized with an overdose of ketamine/xylazine (1:1) and perfused intracardiacally with 4% PFA. The spinal cord was dissected and the lumbar intumescence treated in serial concentrations of sucrose for freezing in O.C.T. Tissue Tek® embedding medium. In a cryostat, cuts were made at a thickness of 12 μm in order to position 4 cuts per slide. A total of 45 slides were mounted, starting at L6 and ending at L4-L3. Immunohistochemistry was performed to identify astrocytes, microglia and to assess demyelination in the white matter. For this purpose, anti-Iba-1 (Wako cat#019–19741, conc. 1:750) and anti-GFAP (Invitrogen cat#13–0300, conc. 1:750) were used as primary antibodies. FluoroMyelin (LifeTechnologies cat# F34652, conc. 1:300) was used to stain myelin (Table 5). The slides were washed 3 times for 5 min each with 0.01 M phosphate-buffered saline (PB) and incubated in a 5% BSA blocking solution for 60 min. After blocking, the sections were incubated in the primary antibodies overnight at 4°C. The following day, the primary antibody was removed and the slides washed again with 0.01 M PB for three times of 5 min each. The secondary antibody was then added for 60 min at room temperature. The slides were washed again and then DAPI at 0.1 mg/mL and FluoroMyelin were added for 10 min. Finally, the slides were mounted with coverslips in glycerol. Three slides of serial sections of the lumbar intumescence of each animal were viewed under a Leica DM5500 B microscope and the images recorded by a DFC345X fluorescence camera. Images of the ventral horn of the spinal cord for the markers GFAP and Iba-1 were evaluated by integrated density of pixels (IDP) using ImageJ software (RRID: SCR_003070). Microglia morphology was also assessed and classified according to Choi et al. (2021) and Reddaway et al. (2023). Demyelination was assessed by comparing the IDP of FluoroMyelin labeling in the white matter by dorsal, lateral and ventral funiculus of animals with EAE and naïve animals. The evaluation by funicle aimed to identify whether there was a prevalence toward areas of demyelination/remyelination in the disease and post-treatment (Supplementary Figure S1). Figure 1 shows the vigilant microglia identified when in branched (I) and hyper-ramified (II) morphology. Reactive microglia, on the other hand, have more retracted branches and a larger cell body (III), or may be amoeboid (IV) and rod-shaped (V).

### 2.12 Statistical analysis

The normality of the data was assessed using the Shapiro–Wilk test. Differences between differentiated NSC and OPC were assessed using Student’s *t*-test. The groups of experimental animals were tested by analysis of variance (ANOVA), followed by Tukey’s test using GraphPad Prism 9.0 software (RRID: SCR_002798). Statistical significance was adjusted for *p*-values ≤0.05. Data is presented as mean ± standard error of the mean.

## 3 Results

### 3.1 Exosomes can be isolated from the conditioned medium of NSC-derived OPCs

**Figure 2:**
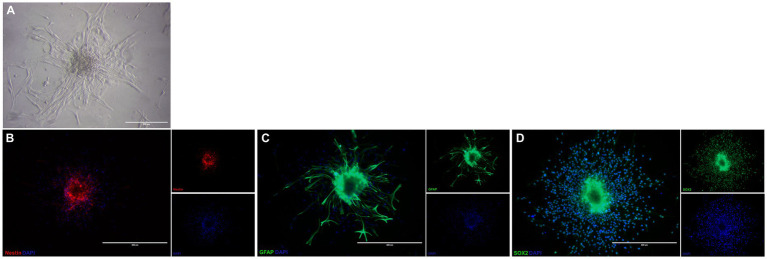
Characterization of neural stem cells isolated from subventricular zone of mice. NSC in treated plate appearing as adherent neurospheres **(A)**. These NSC are immunopositive for Nestin **(B)**, GFAP **(C),** and SOX-2 **(D)**. Scale bar: 400 μm.

**Figure 3:**
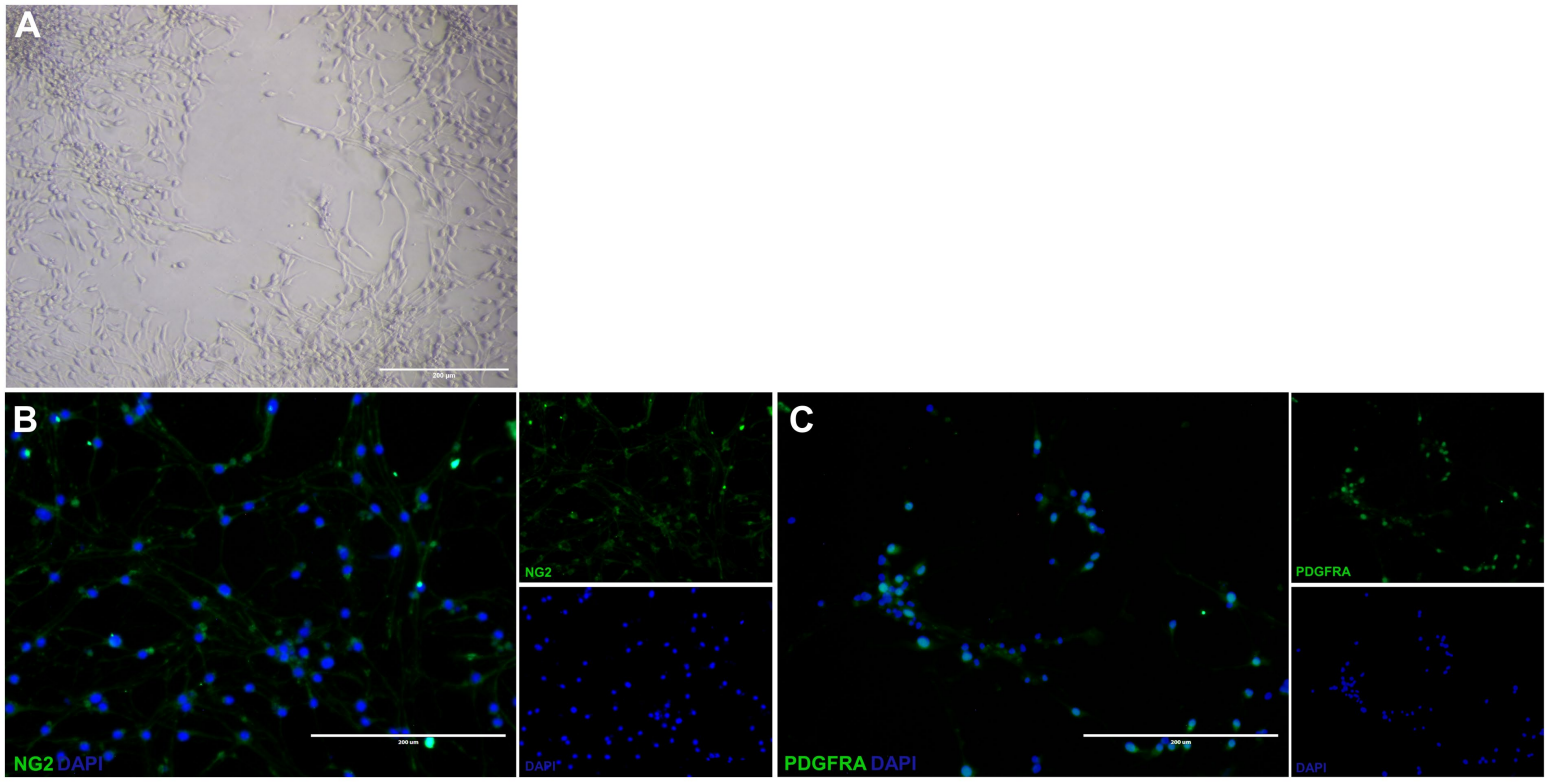
Differentiated oligodendrocyte precursor cells from neural stem cells. Differentiated OPC have a rounded cell body and elongated bipolar processes **(A)**. These OPC is immunopositive for NG2 **(B)** and PDGFRA **(C)**. Scale bar: 400 μm.

**Figure 4:**
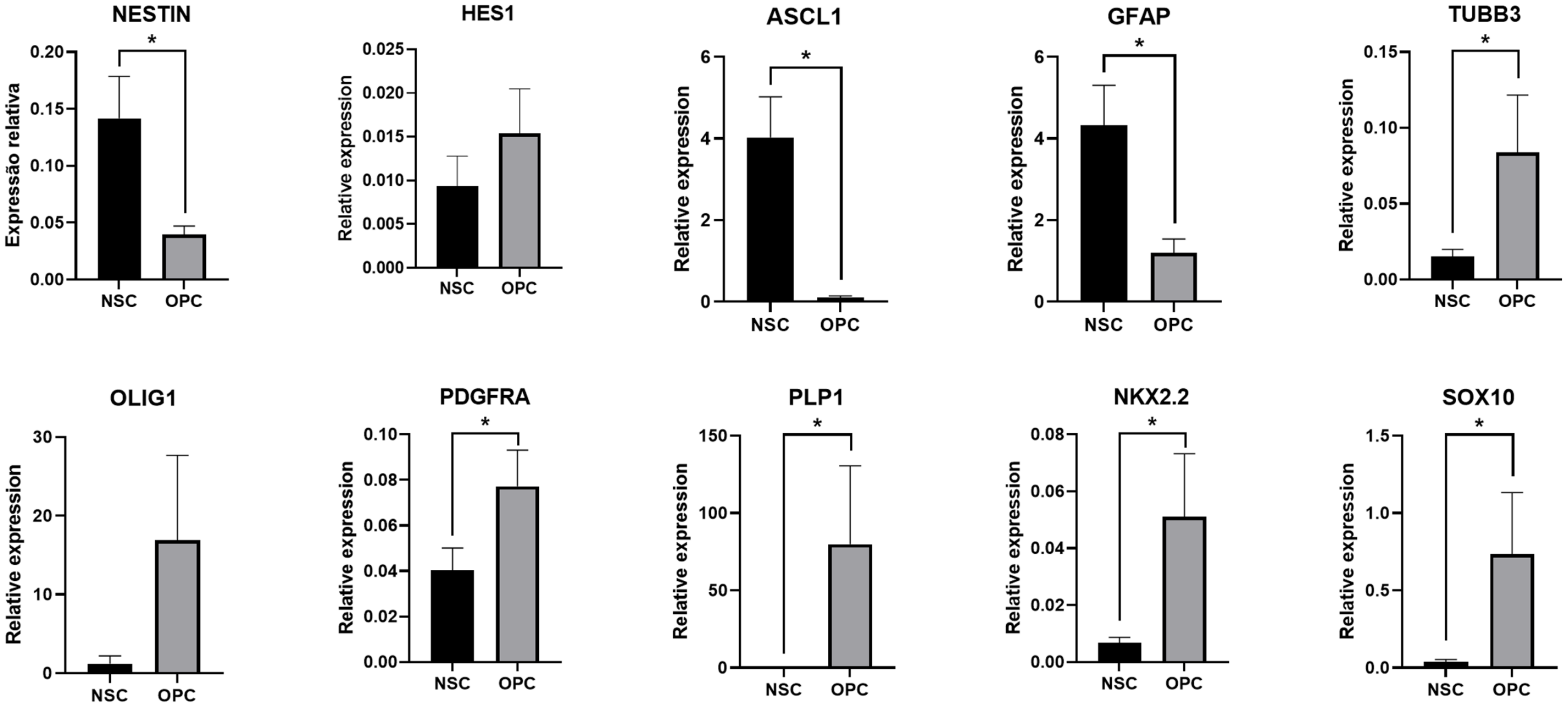
Relative expression shows a decrease in genes related to NSCs, such as *NESTIN*, and an increase in the expression of genes related to the oligodendrocyte lineage. The expression levels of each gene were normalized to the expression of the endogenous GAPDH gene. *p ≤ 0.05.

The cells isolated from the subventricular zone generated neurospheres following 7 days in culture and were re-plated to adherent culture in order to induce differentiation into OPCs and continue with the other tests. NSC were induced to differentiate into OPC for 4 days and changed their morphology from a flat epithelial cell body with multiple processes (Figure 2A) to a morphology typical of OPCs with a rounded cell body and elongated bipolar processes (Figure 3A). The multipotent state of the NSCs and their ability to differentiate into neural lineages was confirmed by positive immunolabeling for Nestin (Figure 2B), GFAP (Figure 2C) and SOX2 (Figure 2D). However, there was no labeling for β-tubulin III, a neuronal biomarker. After differentiation, we used the NG2 (Figure 3B) and PDGFRA (Figure 3C) markers to confirm the phenotype of the OPCs that were positive. We quantified the number of immunopositive cells for each marker in order to determine the homogeneity of our cell population and our results achieved a total of 94.7 ± 3.3% of cells immunopositive for NG2 and 93.6 ± 2.1% of cells immunopositive for PDGFRA after 4 days of the differentiation protocol. We also immunolabeled for a myelin marker MBP that would indicate oligodendrocyte maturation and it was immunonegative in the OPCs (Supplementary Figure S2). By analyzing gene expression, NSCs expressed high levels of *NESTIN*, which decreased considerably after differentiation into OPCs. The expression levels of genes related to the differentiation process in oligodendrocytes reaffirmed the success of the induced cell differentiation, since the levels of *PDGFRA, NKX2.2, PLP1* and *SOX10* are low in NSCs and significantly increased in OPCs derived from these cells. *ASCL1* was also significantly reduced in OPC and this gene is related to the process of cell differentiation (Figure 4).

**Figure 5:**
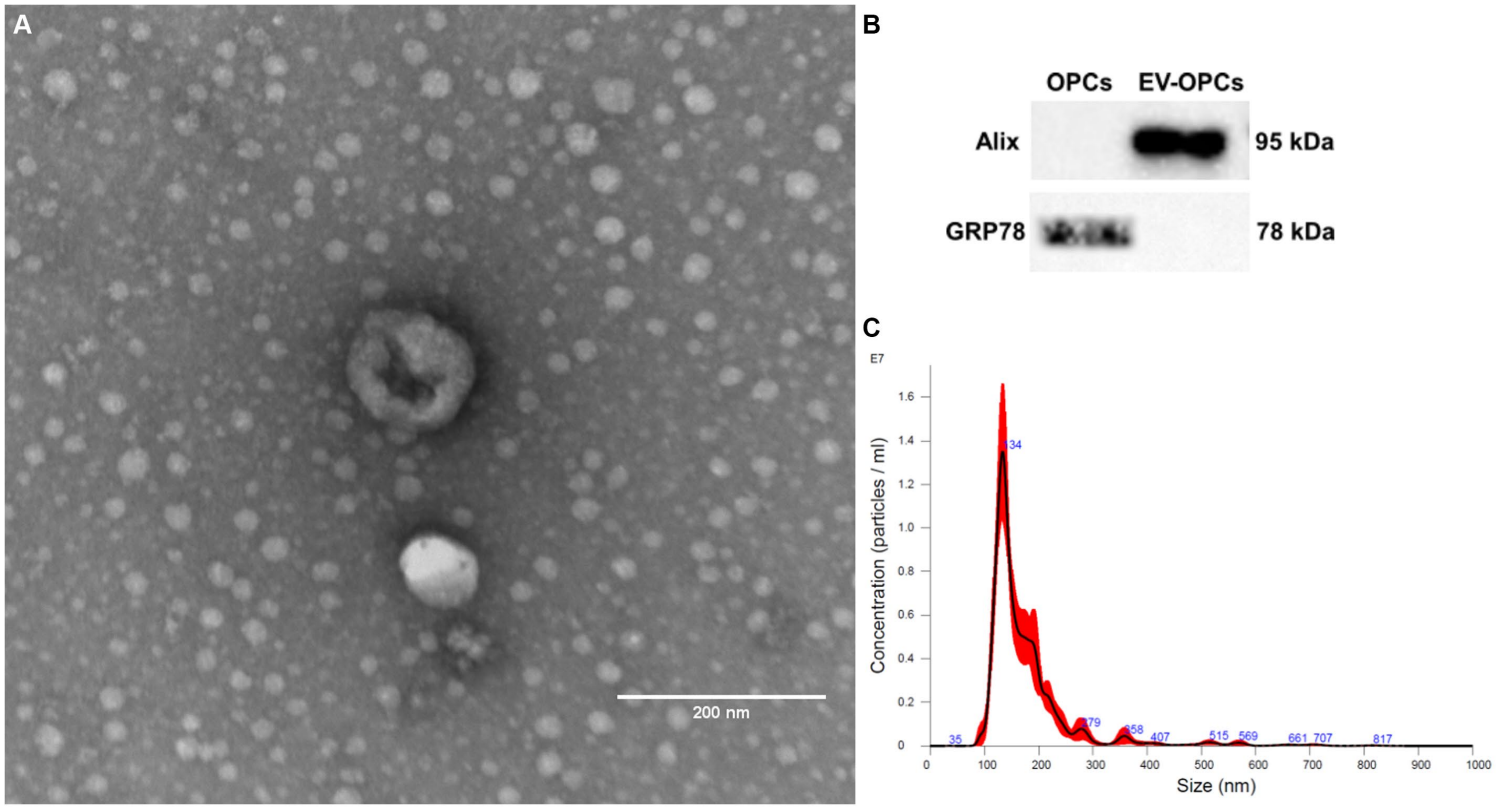
Characterization of OPC-derived exosomes by transmission electron microscopy **(A)**, western blotting **(B)** and nanoparticle tracking analysis **(C)**. Scale bar: 200 nm.

EV-OPC isolated from the conditioned medium of 75 cm^3^ flasks with 80% cell confluence were studied by NTA. The average size of the exosomes was 155.2 ± 9.04 nm and the concentration were 2.24 ± 0.54 × 10^8^ particles/mL. Figure 5C shows the homogeneity in the size of extracellular vesicles by NTA. Transmission electron microscopy allowed the morphology of EV-OPC to be viewed and distinguished from other particles. Here, EVs can be identified by their size and cup-shaped morphology (Figure 5A). EVs should also be characterized by the detection of proteins specific to these structures, and the purity of their isolation can also be verified by the absence of proteins common to cell organelles. Using Western blotting method, the endoplasmic reticulum protein GRP78 was detected in OPC lysate, which was absent in the EV-OPC lysate. The exosomal protein Alix was detected in the EV-OPC lysate but not in the cell lysate revealing the purity of isolation (Figure 5B).

### 3.2 EV-OPCs promote clinical recovery of EAE

**Figure 6:**
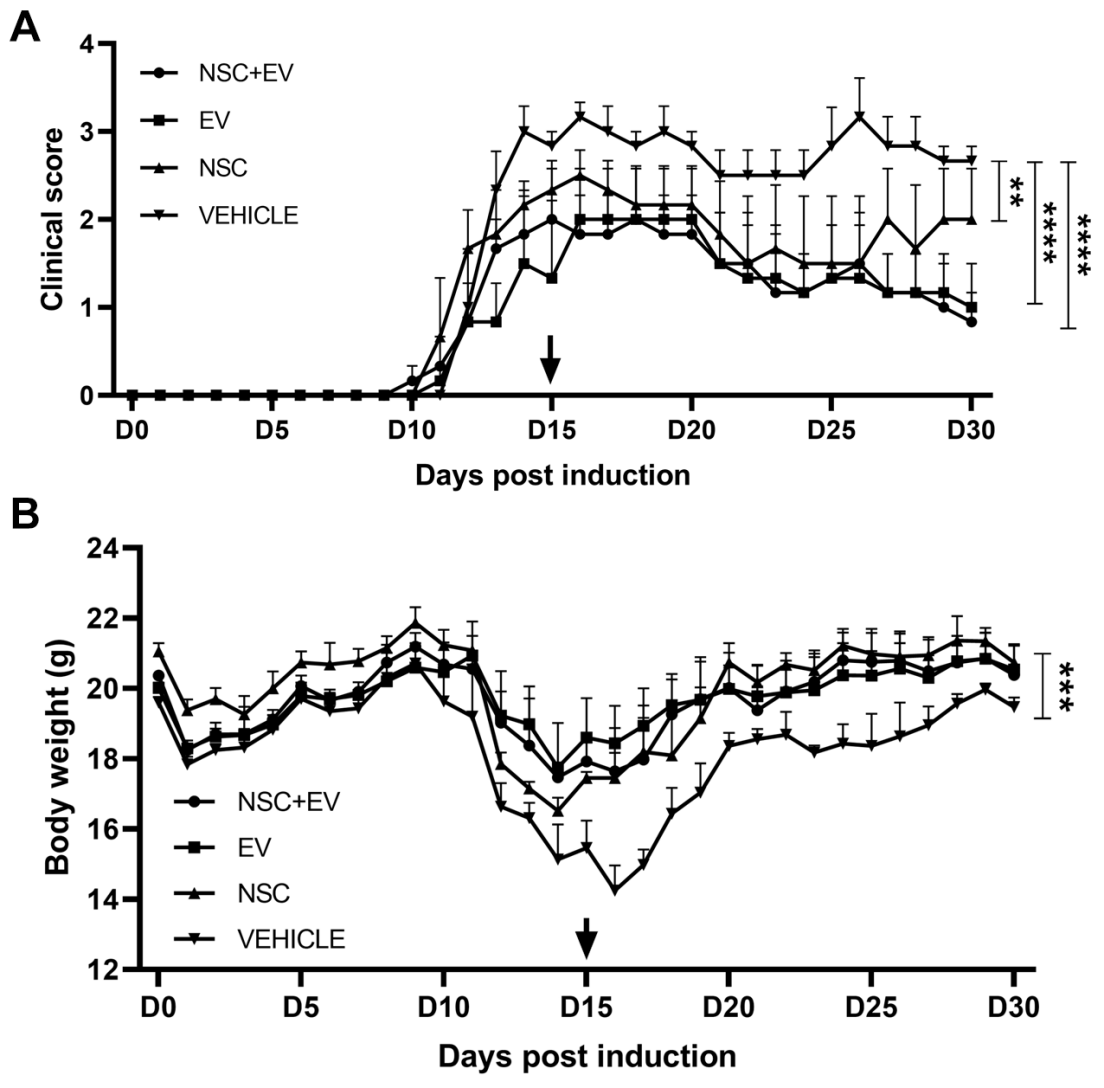
Clinical score assessment and body weight of EAE mice groups treated with NSC + EV, EV and NSC compared to EAE vehicle control group. All groups presented significant differences in clinical score and body weight compared to control group. Clinical score of NSC + EV and EV groups had *p* ≤ 0.0001 while NSC group had *p* ≤ 0.01 **(A)**. Body weight of NSC + EV, EV and NSC groups had *p* ≤ 0.001 **(B).** The *p*-value was determined by the ANOVA test followed by Tukey’s test.

The animals had their clinical score and weight measured daily. The vehicle-only (PBS) control group had a stable clinical score with two disease peaks. The mean EAE scores in the control group (2.7 ± 0.05) were significantly higher than those in the groups treated with NSC (1.9 ± 0.08), EV (1.4 ± 0.09) and co-treatment of NSC + EV (1.4 ± 0.09). Although the graph shows a late second peak in the group treated with NSC, while the groups receiving EVs did not show this second peak, there were no significant differences between treatments. Weight loss started 2 days before the onset of clinical signs in all groups and followed the clinical evolution of the animals. Weight loss occurred as the clinical score increased and weight gain occurred as the clinical score recovered. Weight loss in the vehicle control group (17.84 g ± 0.42) was significantly higher than in the groups treated with NSC (20.0 g ± 0.35), EV (19.91 g ± 0.18) and the co-treatment of NSC + EV (19.8 g ± 0.27), which showed no differences between them (Figure 6).

### 3.3 EV-OPCs modulates the profile of Th1 cells to Th2 cells

**Figure 7:**
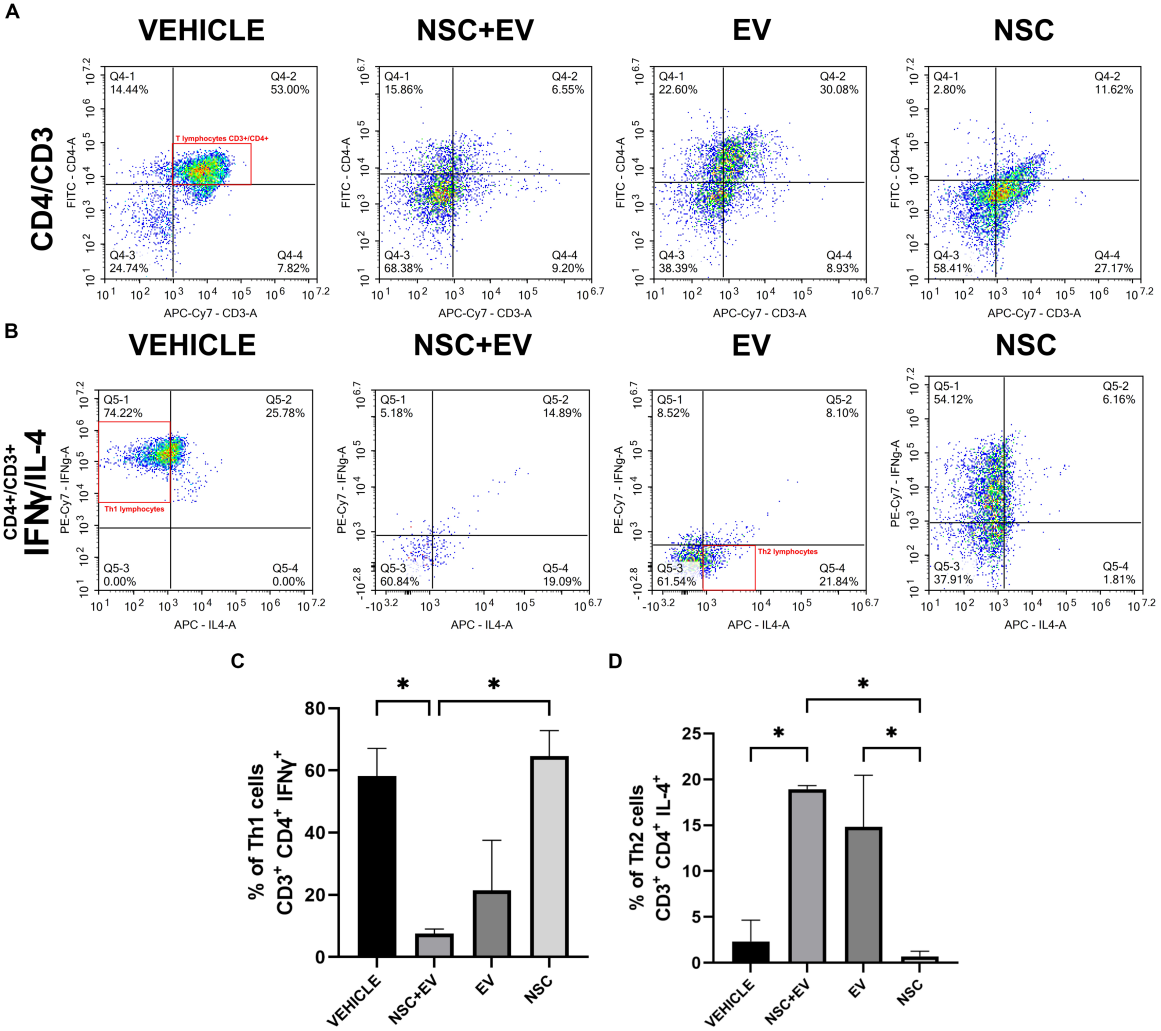
Flow cytometry of spinal cord cells from EAE animals. Dot-plot of the events analyzed for CD3 and CD4 markers presents the amount of double-labeled cells **(A)** that were then reanalyzed for the cytokines IFNγ and IL-4 **(B)**. The results were organized in graphs showing a decrease in cells with a Th1 profile **(C)** and an increase in Th2 cells in the animals treated with NSC + EV and EV **(D).** *p ≤ 0.05.

The lymphocyte phenotype study aimed to identify the pro-inflammatory or anti-inflammatory profile of these cells in the spinal cord of EAE treated and control animals. Flow cytometry was used to identify each cell type by surface markers and cytokines produced. CD4+ T lymphocytes were identified by double CD3+ and CD4+ labeling. Th1 lymphocytes were identified by IFNγ labeling and Th2 lymphocytes by IL-4 labeling (Figures 7A,B). The proportion of Th1 or neuroinflammatory cells were significantly lower in the NSC + EV group compared to the vehicle control and NSC-only groups. Besides Th2 or neuroprotective cells was significantly higher in the two groups receiving EVs in comparison to the control and NSC groups (Figures 7C,D). This result suggests a modulation of CD4+ T lymphocytes promoted by EV-OPCs.

### 3.4 EV-OPCs attenuate gliosis alone or in combination with cell therapy

**Figure 8:**
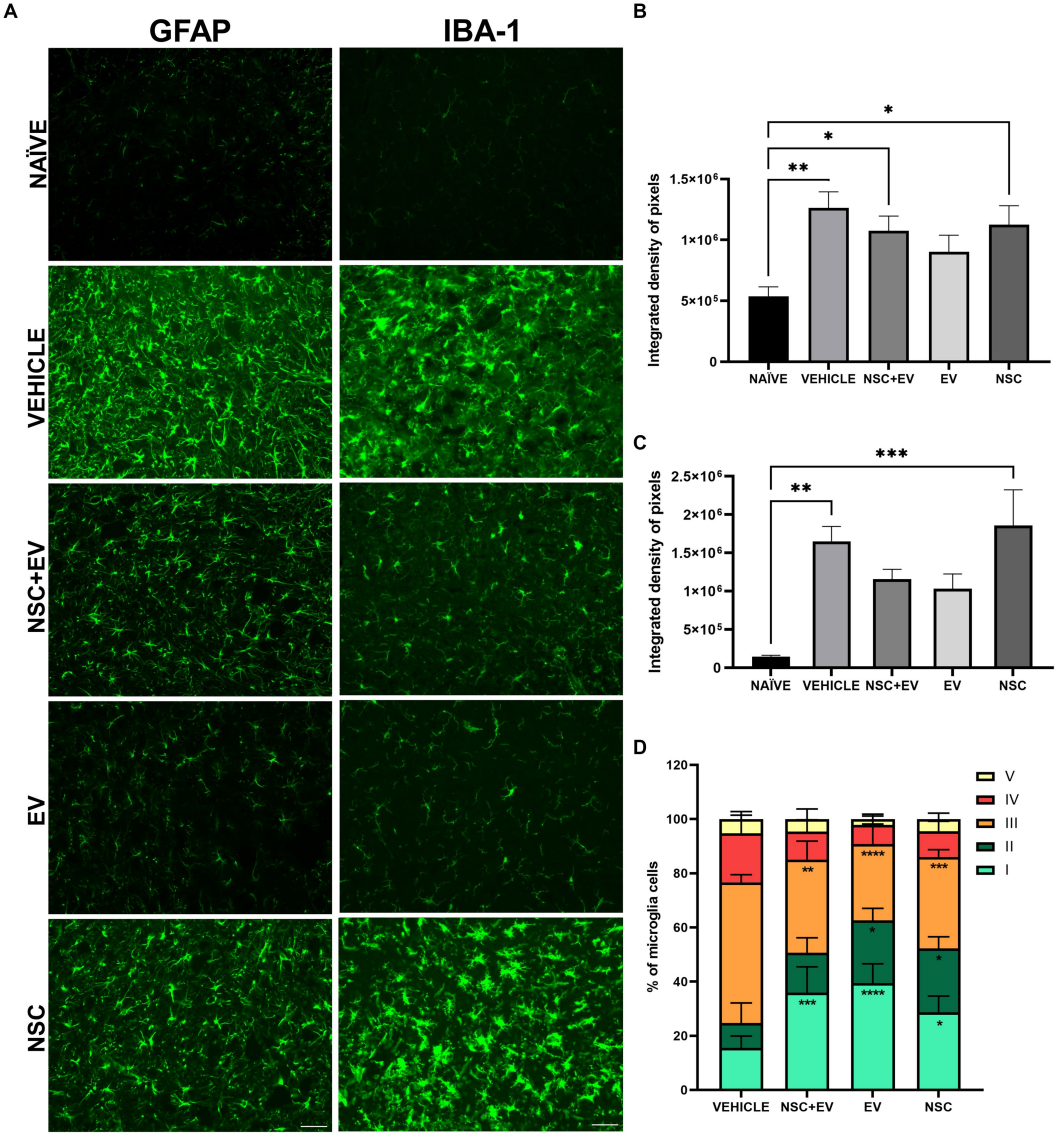
Immunohistochemistry for GFAP and Iba-1 markers in the spinal cord of naive and EAE animals **(A)**. The quantification of IDP for GFAP demonstrates a reduction in the proportion of astrocytes in the ventral horns since there is no significant difference between the EV group and the naive animals **(B)**. IDP for Iba-1 shows a reduction in the presence of microglia in both groups treated with EVs, as there was no difference between the groups and the naive animals **(C)**. The microglia classification shows that the amount of reactive microglia was lower in all treated groups when compared to the vehicle control group **(D)**. *p ≤ 0.05, **p ≤ 0.01, ***p ≤ 0.001, ****p ≤ 0.0001. Scale bar: 50 μm.

The immunohistochemical analysis of some markers allowed a better understanding of the morphocellular events in the spinal cord promoted not only by the induction of EAE, but also by the post-induction treatment (Figure 8A). GFAP immunolabelling, used to identify astrocytes, was shown to be significantly higher in EAE-induced mice compared to naïve mice. This was also found for the control animals, as well as for the groups that received NSCs. On the other hand, there were no differences between the naïve animals and the EV-treated group, showing a promising result in EV-OPCs attenuating astrogliosis (Figure 8B). Immunolabelling for Iba-1 allowed assessment of the distribution and morphology of microglia in naïve and EAE animals. The prevalence of microglias was significantly higher in the induced EAE animals treated with vehicle and NSC group. However, there were no statistical differences between the healthy animals and both groups that received EV-OPCs, demonstrating the relevance of exosomes for this result (Figure 8C). When morphology was investigated, all EAE-treated groups showed significant differences in the proportion of vigilant and reactive microglia compared to the control group. Vigilant (or homeostatic) microglia was higher in the treated groups, whereas reactive microglia were significantly lower compared to EAE control (Tables 4–5; Figure 8D).

**Table 4:** Antibodies used for spinal cord flow cytometry.

**Antibodies**	**Fluorophore**	**Source (RRID)**	**Dilution**
Live/Dead	Chemical	Invitrogen (L34968A)	1:100
GFAP	Alexa Fluor 488	BioLegend (AB_2566109)	1:100
IL-4	APC	BioLegend (AB_315320)	1:50
IL-10	PE	BioLegend (AB_315362)	1:50
IFNγ	PE/Cyanine7	BioLegend (AB_2295770)	1:50
TNFα	PerCP/Cyanine5.5	BioLegend (AB_961434)	1:50
CD68	APC	BioLegend (AB_10575299)	1:50
CD206	PE/Cyanine7	BioLegend (AB_2562248)	1:50
CD3	APC/Cyanine7	BioLegend (AB_2057374)	1:50
CD4	Alexa Fluor 488	BioLegend (AB_389302)	1:50
CD45	Alexa Fluor 488	BioLegend (AB_493532)	1:50
CD11b	APC/Cyanine7	BioLegend (AB_830641)	1:50

**Table 5:** Antibodies used for spinal cord immunohistochemistry.

**Antibodies**	**Host**	**Source (RRID)**	**Dilution**
Iba-1	Rabbit	Wako (AB_839504)	1:750
GFAP	Rat	Invitrogen (AB_2532994)	1:750
FluoroMyelin	Chemical	LifeTechnologies (AB_2572213)	1:300
Alexa488	Rat	Invitrogen (AB_1500699)	1:250
Alexa488	Rabbit	Invitrogen (AB_2535711)	1:250

### 3.5 Microglial infiltrate and demyelination are less apparent in EAE-treated animals

**Figure 9:**
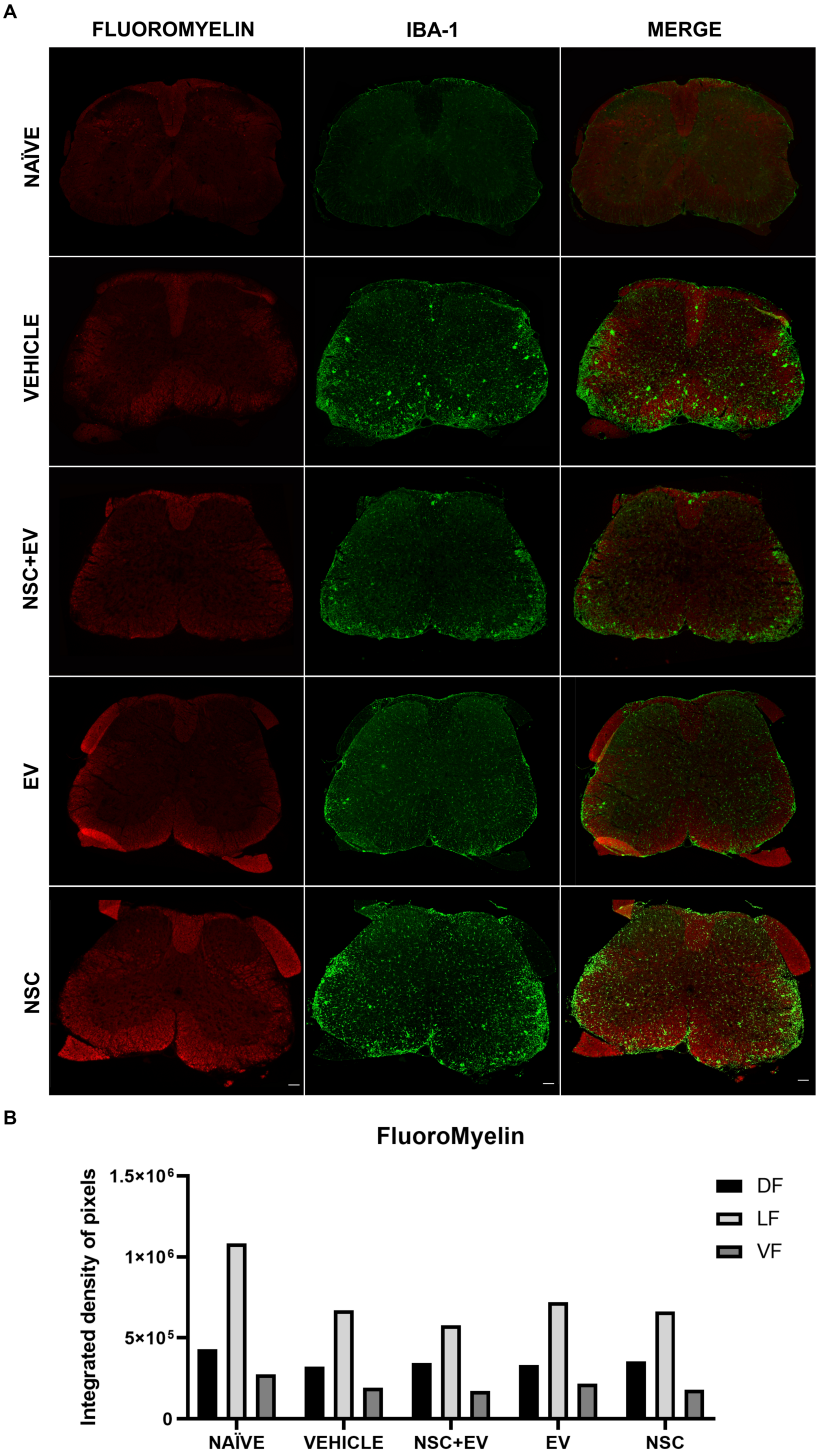
Demyelination followed by microglial infiltration in the white matter is evident in all EAE groups **(A)**. However, IDP quantification of the funiculi showed no significant differences between the groups **(B)**. Scale bar: 100 μm.

FluoroMyelin was used as a myelin marker to detect areas of demyelination and quantify IDP for labeling in the dorsal (DF), lateral (LF) and ventral (VF) funiculus. The areas with loss of red staining (demyelination) in the white matter are proportionally invaded by microglia identified by the Iba-1 marker. These areas of demyelination with large populations of microglia are more prominent in the control EAE group than in the treated and naïve groups (Figure 9A). However, quantification of IDP showed no significant differences between groups (Figure 9B).

## 4 Discussion

NSCs in this study were immunopositive for nestin, SOX-2 and GFAP, but there was no labeling for β-tubulin III. Although the presence of β-tubulin III+ cells in NSC culture has been reported (Cheng et al., 2016; Lee et al., 2020), this protein can also be absent (Micci et al., 2019). Supplementing the culture medium with B27 without vitamin A (retinol acetate) may have affected the disposition of β-tubulin III, as retinol acetate metabolites are essential for differentiation and maintenance of the neuronal lineage (Lu et al., 2009; Chen et al., 2022).

The differentiation protocol of Li et al. (2019), used as a method to differentiate NSC into OPC, describes a fast and efficient protocol by supplementing the culture medium with SAG, bFGF and PDGFA. This supplementation induces the activation of the Sonic Hedgehog (Shh) signaling pathway, which is critical for the development and proliferation of OPCs (Hu et al., 2012; Ferent et al., 2013). Following this protocol, rounded cell body with typical bipolar or tripolar morphology were obtained as observed by Li et al. (2019) and other studies (Yang et al., 2016). In addition, differentiated cells were immunopositive for NG2 and PDGFRA, specific markers of this cell type (Li et al., 2017). While the maturation marker MBP was immunonegative, which is expected for this type of precursor cell (Lourenço et al., 2016). The results of the gene expression also corroborate the phenotype found in the differentiated OPC cells (Otsu et al., 2021; Figures 7–9).

In experimental autoimmune encephalomyelitis, the choice of animal and method of sensitization is crucial for achieving the expected clinical and pathological characteristics, as they aim to mimic different aspects of multiple sclerosis (Ballerini, 2021). In the model using C57BL/6 mice sensitized with MOG35-55, the animals lose weight 1 to 2 days before the first clinical signs appear between 9 and 14 dpi (Bittner et al., 2014). In this study, the animals exhibited a consistent pattern, with clinical signs appearing around 11 dpi and the peak of disease around 16 dpi similar to the optimized protocol of Huntemann et al. (2022). The treatment was initiated on day 15 post induction, when all animals were clinically ill, to assess whether the treatments would be effective in reversing or ameliorating the course of the disease.

The study investigated the role of EVs in optimizing cell therapy by treating the animals with NSC, EV-OPC and their combination. Weight gain and improvement in clinical signs are expected after the peak of the disease because the model mimics the remitting and relapsing aspects of multiple sclerosis (Huntemann et al., 2022). However, all experimental groups showed significant clinical improvements and weight gain compared to the control. Promising clinical results have been achieved in several studies using neural stem cells for EAE therapy, as demonstrated by Wu (2013), Nasri et al. (2018), and Brown et al. (2021). The use of EVs, however, is advancing rapidly as they are biocompatible nanoparticles and an alternative to regenerative cell therapy. Stem cell-derived EVs are often the most widely used in experimental therapies due to the paracrine substances that modulate the immune system and induce regeneration (Kou et al., 2022). The transplantation of OPCs for the treatment of central nervous disorders has shown promising results with the recovery of motor function, improvement of neurite growth and synaptogenesis (Sun et al., 2013; Wang et al., 2020). Conversely, until now OPC-derived EVs have never been used in animal therapy and little is known about the biomolecules they contain. In EAE studies, there are several applications of EVs with different dosages and important results, where the concentration can be described in μg of protein or particles/mL (Casella et al., 2018; Fathollahi et al., 2021; Gupta et al., 2021). In this study, a single dose of EV-OPCs at a concentration of 2.5 × 10^9^ particles/mL was effective in improving the clinical outcome of both treatment groups. A single intravenous dose of EVs in EAE mice is effective at a high concentration and is not clinically effective at a lower concentration (Clark et al., 2019) or at low concentrations applied in multiple doses (Giunti et al., 2021). As for NSCs, the concentration of 5 × 10^5^ cells/mL was effective in improving the clinical condition of the animals and led to a reduction in the proportion of reactive microglia. Still, there was no modulation of CD4+ T lymphocytes or reduction in astrogliosis, as observed in the groups treated with EVs. Additionally, even at higher doses, the application of 1 × 10^6^ cells in EAE animals triggered a reduction in astrocytes only in the brain, but not in the spinal cord (Brown et al., 2021). In both groups treated with EVs, there was a decrease in the proportion of Th1-type CD4+ T lymphocytes and an increase in Th2 lymphocytes in the spinal cord. The same response was not seen in the group treated with cells alone. This indicates that the modulation of CD4+ T lymphocytes was promoted by EVs. In EAE, Th1 and Th17 lymphocytes are widely known to mediate the onset, progression and relapse of the disease, while Th2 lymphocytes are responsible for disease remission (Fernando et al., 2014). Th2 lymphocytes produce the cytokine IL-4 and IL-10, which are associated with protective effects in various pathologies of the nervous system, including EAE. These effects include suppression of antigen-presenting cells and inhibition of Th1 lymphocytes (Rasquinha et al., 2021; Mamuladze and Kipnis, 2023). In EAE mice treated with IL-4-enriched EVs, the animals showed significant clinical improvement and modulation of phagocytes toward an anti-inflammatory phenotype (Casella et al., 2018).

We evaluate some of the pathological features of EAE by immunohistochemistry. GFAP marker revealed a significant reduction in astrocytosis in the animals treated with EVs. Similarly, the Iba-1 marker showed a reduction in reactive microgliosis, both in general quantification and in the study of microglia morphology. Both treatment groups receiving EVs showed similar clinical improvement, possibly due to the attenuation of gliosis, which was also similar between them. However, the cell-only group showed a second peak in the clinical score, despite clinical improvement, due to factors that were not elucidated by our analysis. Nevertheless, the difference in improvement and the appearance of a second peak confirms that the clinical effects of treatment with cells and EVs follow different pathways. In EAE, astrocytes and microglia become reactive in response to Th1 and Th17 cell stimuli that activate the secretion of pro-inflammatory cytokines such as TNFα, IL-6 and IL-1β and reduce the secretion of neurotrophic factors by astrocytes such as NGF, CNTF and BDNF (Prajeeth et al., 2017; Krishnarajah and Becher, 2022). Due to this mechanism, therapeutic strategies in EAE seek to suppress the response of Th1 and Th17 lymphocytes and promote a shift to the Th2 cell phenotype, as well as increase the amount of regulatory T cells (Kunkl et al., 2022). By reducing the proportion of Th1 lymphocytes and increasing that of Th2 lymphocytes, treatments with EVs achieved the necessary effect for an attenuation of gliosis. Despite this, in the cell-only group, there was no immunomodulation of the Th1 profile and consequently no attenuation of gliosis. The clinical response of the two groups receiving EVs as a therapeutic treatment was similar. However, the group that received EVs only showed slightly better results than the NSC + EV combination group in terms of gliosis and lymphocyte response. Our analyses did not reveal the reason for this. However, a limiting factor in cell therapy is the patient’s immunogenic response to cell transplantation. Although neural stem cells have low immunogenicity, they can produce reactive gliosis a few days after treatment, which tends to resolve within a few weeks (Wei et al., 2021). Alternatively, EVs offer almost irrelevant immunogenicity (Zhu et al., 2017) and have been shown to be lower than cell therapy (Zheng et al., 2018). It is possible that the use of cells in combination with EVs provided an immunogenic response that made the combination less interesting than treatment with EVs only.

Demyelination was accompanied by microglial infiltration in the EAE animals, it can be seen that in the control animals, this microglial infiltrate in the sites of demyelination is more apparent than in the animals treated with EVs. Nevertheless, the DIP quantification method for FluoroMyelin in the white matter showed no significant differences even between the healthy animals and the control group. The demyelination of the animals in this study was assessed at a time when all the animals, especially the control group, were in the remission phase of the clinical score. Although remyelination was one of the expected processes involved in motor recovery, the attenuation of gliosis in the ventral horn appears to have recovered it in the treated animals. In a study by Casella et al. (2020), EVs derived from mature oligodendrocytes were used in the therapeutic treatment of EAE and improved the course of the disease. This improvement was associated with a decrease in Th1 cells and an increase in the number of regulatory T lymphocytes as a result of tolerance induced by myelin proteins that act as antigens and are present in these EVs. Yet, they found that the myelin proteins MOG, MBP and PLP, which are responsible for the induced tolerance and clinical response of the animals, are absent in the EV-OPCs. Despite EV-OPCs are also able to interact with the immune system and modulate this response in Th1 cells, the mechanisms of this interaction are unknown since little is known about the biomolecule content of EV-OPCs and which could be responsible for the effects observed here. Therefore, further studies are essential to understand the potential of these EVs in EAE therapy.

## Data availability statement

The datasets presented in this study can be found in online repositories. The names of the repository/repositories and accession number(s) can be found in the article/Supplementary material.

## Ethics statement

The animal study was approved by Comissão de Ética no Uso de Animais (CEUA) – University of São Paulo and State University of Campinas. The study was conducted in accordance with the local legislation and institutional requirements.

## Author contributions

SS: Conceptualization, Data curation, Formal analysis, Funding acquisition, Investigation, Methodology, Project administration, Resources, Software, Supervision, Validation, Visualization, Writing – original draft, Writing – review & editing. SO-P: Conceptualization, Data curation, Formal analysis, Investigation, Methodology, Project administration, Software, Writing – original draft, Writing – review & editing. AP: Data curation, Formal analysis, Methodology, Validation, Writing – original draft, Writing – review & editing. AT: Data curation, Formal analysis, Methodology, Validation, Writing – original draft, Writing – review & editing. LC: Data curation, Formal analysis, Methodology, Validation, Writing – original draft, Writing – review & editing. JS: Conceptualization, Formal analysis, Investigation, Methodology, Resources, Software, Visualization, Writing – original draft, Writing – review & editing. DM: Funding acquisition, Methodology, Resources, Writing – original draft, Writing – review & editing. AC: Methodology, Resources, Writing – original draft, Writing – review & editing. AO: Conceptualization, Investigation, Methodology, Resources, Software, Supervision, Validation, Writing – original draft, Writing – review & editing. CA: Funding acquisition, Investigation, Project administration, Resources, Supervision, Writing – original draft, Writing – review & editing.
