# Corrigendum: Oligodendrocyte precursor cell-derived exosomes combined with cell therapy promote clinical recovery by immunomodulation and gliosis attenuation

**DOI:** 10.3389/fncel.2024.1546979

**Published:** 2025-01-07

**Authors:** Sarah Ingrid Pinto Santos, Santiago José Ortiz-Peñuela, Alessandro de Paula Filho, Ana Laura Midori Rossi Tomiyama, Lilian de Oliveira Coser, Juliano Coelho da Silveira, Daniele dos Santos Martins, Adriano Polican Ciena, Alexandre Leite Rodrigues de Oliveira, Carlos Eduardo Ambrósio

**Affiliations:** ^1^Faculty of Animal Science and Food Engineering, University of São Paulo (FZEA/USP), São Paulo, Brazil; ^2^Institute of Biology, University of Campinas (IB/UNICAMP), Campinas, Brazil; ^3^Institute of Biosciences, São Paulo State University, Rio Claro, Brazil

**Keywords:** extracellular vesicles, multiple sclerosis, neural stem cell, neurodegeneration, immunomodulation, oligodendrocyte precursor cell

In the published article, there was an error in the **Funding statement**. In the Funding statement for the São Paulo Research Foundation, only grant 2021/09869–6 was displayed. The correct statement is São Paulo Research Foundation grant 2021/09869–6 and grant 2022/10604-0. The correct Funding statement appears below.

