# Supplementary material for: Oligodendrocyte precursor cell-derived exosomes combined with cell therapy promote clinical recovery by immunomodulation and gliosis attenuation
Source: Front Cell Neurosci. 2024 Jul 23;18:1413843. doi: 10.3389/fncel.2024.1413843 (PMC11301646; doi:10.3389/fncel.2024.1413843)
Supplement: Supplementary file 1 [file Data_Sheet_1.DOCX]

Supplementary Material

Oligodendrocyte precursor cell-derived exosomes combined with cell therapy promote clinical recovery by immunomodulation and gliosis attenuation

Sarah Ingrid Pinto Santos^1^, Santiago Jose Ortiz Peñuela^2^, Alessandro de Paula Filho^1^, Ana Laura Midori Rossi Tomiyama^2^, Lilian de Oliveira Coser^2^, Juliano Coelho da Silveira^1^, Daniele dos Santos Martins^1^, Adriano Polican Ciena^3^, Alexandre Leite Rodrigues de Oliveira^2^, Carlos Eduardo Ambrósio^1^

^1^Faculty of Animal Science and Food Engineering, University of São Paulo (FZEA/USP), Brazil

^2^Institute of Biology, University of Campinas (IB/UNICAMP), Brazil

^3^Institute of Biosciences, São Paulo State University, Campus Rio Claro (IB/Unesp), Brazil

*** Correspondence:**Corresponding Author
sarahingridpsantos@gmail.com

**
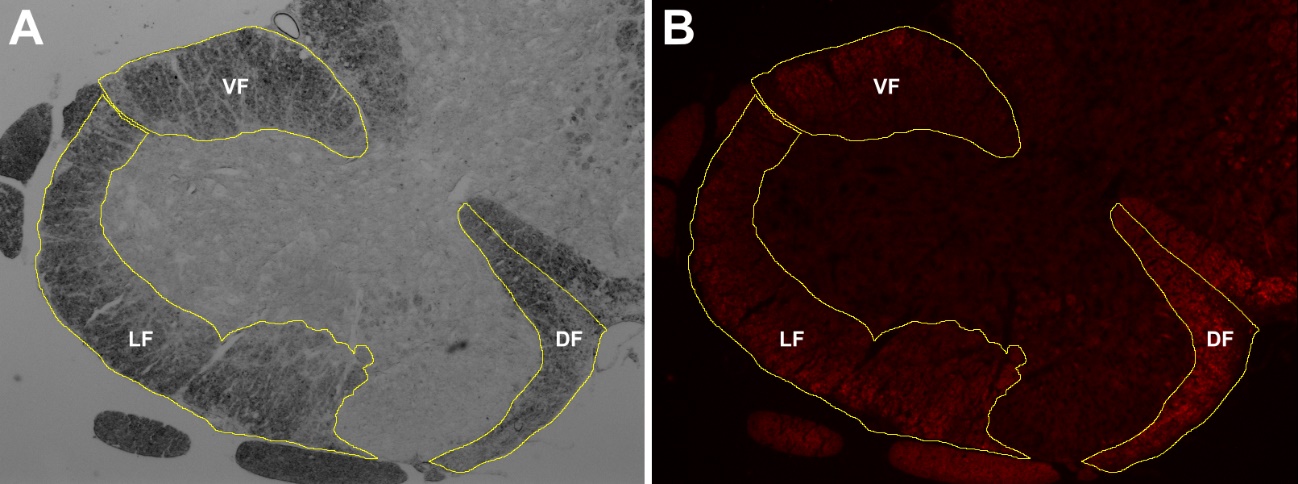
**

**Supplementary figure 1.** Analysis of areas of demyelination in the spinal cord. The area of white matter is initially delineated in the light microscopy image (A) and transferred to the FluoroMyelin-labeled fluorescence image of the spinal cord (B) for subsequent quantification of the integrated pixel density per funiculus.

**
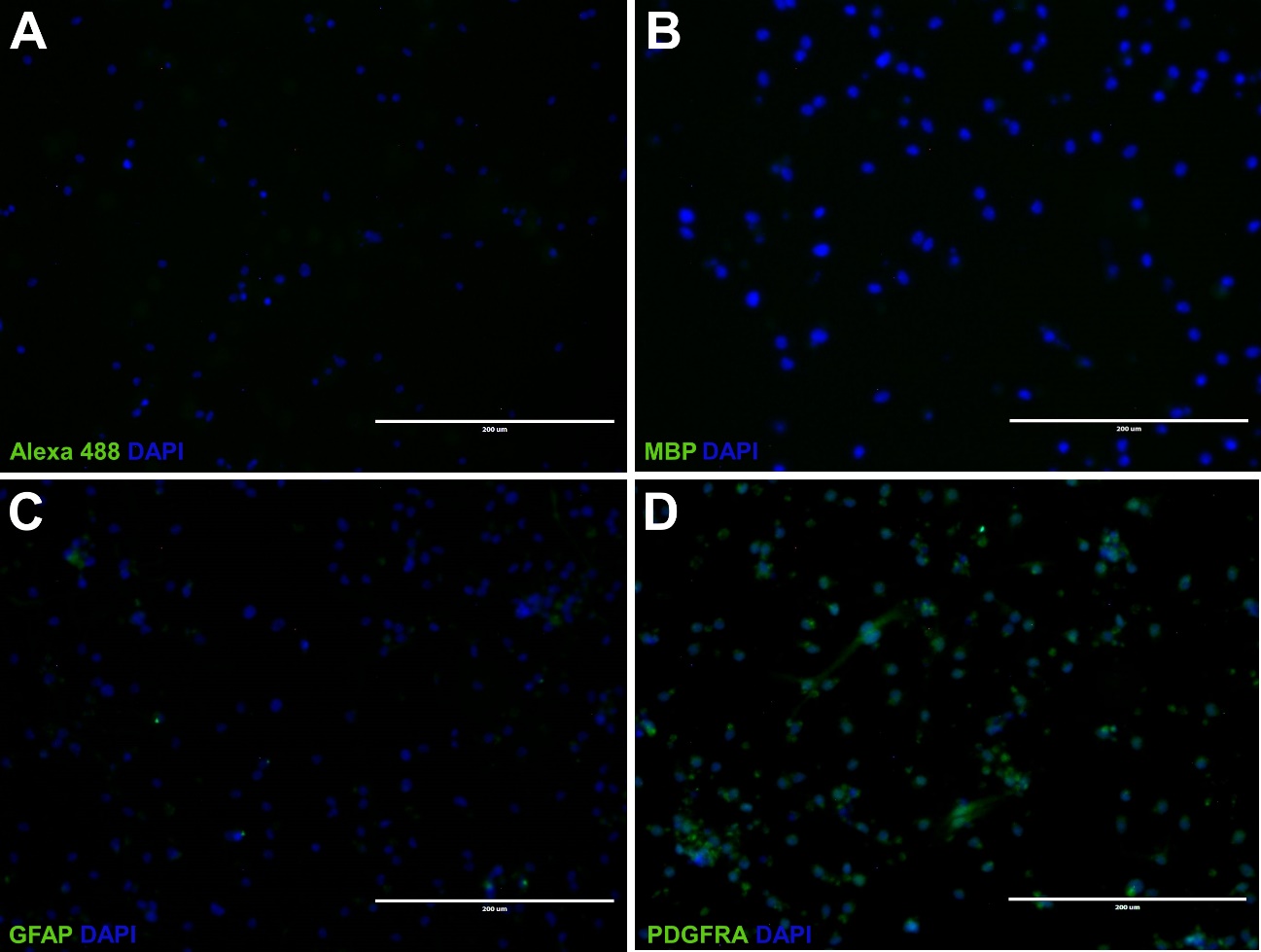
**

**Supplementary figure 2.** Immunocytochemical of differentiated oligodendrocyte precursor cells revealing no labeling in the control cells for the secondary antibody **(A)** and the maturation marker MBP **(B)**. A few cells showed some GFAP labeling **(C)**, while practically the entire population was immunopositive for PDGFRA **(D)**.
